# Reduced vmPFC-insula functional connectivity in generalized anxiety disorder: a Bayesian confirmation study

**DOI:** 10.1038/s41598-023-35939-2

**Published:** 2023-06-14

**Authors:** Jonas L. Steinhäuser, Adam R. Teed, Obada Al-Zoubi, René Hurlemann, Gang Chen, Sahib S. Khalsa

**Affiliations:** 1 Laureate Institute for Brain Research, Tulsa, OK USA; 2 Division of Psychological and Social Medicine and Developmental Neurosciences, Faculty of Medicine, Technische Universität Dresden, Dresden, Germany; 3 Department of Electrical and Computer Engineering, University of Oklahoma, Tulsa, OK USA; 4 Department of Psychiatry, School of Medicine & Health Sciences, University of Oldenburg, Oldenburg, Germany; 5 Research Center Neurosensory Science, University of Oldenburg, Oldenburg, Germany; 6 Scientific and Statistical Computing Core, National Institute of Mental Health, Bethesda, MD USA; 7 Oxley College of Health Sciences, University of Tulsa, Tulsa, OK USA

**Keywords:** Neuroscience, Neural circuits, Psychiatric disorders, Anxiety

## Abstract

Differences in the correlated activity of networked brain regions have been reported in individuals with generalized anxiety disorder (GAD) but an overreliance on null-hypothesis significance testing (NHST) limits the identification of disorder-relevant relationships. In this preregistered study, we applied both a Bayesian statistical framework and NHST to the analysis of resting-state fMRI scans from females with GAD and matched healthy comparison females. Eleven *a-priori* hypotheses about functional connectivity (FC) were evaluated using Bayesian (multilevel model) and frequentist (*t*-test) inference. Reduced FC between the ventromedial prefrontal cortex (vmPFC) and the posterior-mid insula (PMI) was confirmed by both statistical approaches and was associated with anxiety sensitivity. FC between the vmPFC-anterior insula, the amygdala-PMI, and the amygdala-dorsolateral prefrontal cortex (dlPFC) region pairs did not survive multiple comparison correction using the frequentist approach. However, the Bayesian model provided evidence for these region pairs having decreased FC in the GAD group. Leveraging Bayesian modeling, we demonstrate decreased FC of the vmPFC, insula, amygdala, and dlPFC in females with GAD. Exploiting the Bayesian framework revealed FC abnormalities between region pairs excluded by the frequentist analysis and other previously undescribed regions in GAD, demonstrating the value of applying this approach to resting-state FC data in clinical investigations.

## Introduction

Generalized anxiety disorder (GAD) is a psychiatric disorder characterized by disproportionate and uncontrollable worry in addition to somatic symptoms including muscle tension, sleep disturbances, fatigue, and difficulty concentrating. It is a common anxiety disorder, and is associated with substantial functional impairments and economic costs as well as high rates of comorbidity with other psychiatric disorders^1^. While the neurobiology of GAD has been investigated extensively^2^, technical advancements in functional neuroimaging in recent decades have afforded insights into abnormalities of regional and network-level neural communication underlying this condition^3^. Results from many imaging studies suggest that brain regions are organized in distinguishable networks that facilitate complex cognitive functions^4^. Given the aforementioned functional impairments in GAD it is conceivable that these networks (or the nodes within them) are dysfunctional as well^5,6^. Among the most frequently described neural networks are the default mode network (DMN, active during the absence of a specific task)^7^, the salience network (SN, responsible for shifting attention to behaviorally relevant internal and external stimuli)^8^, and the central executive network (CEN, involved in cognitively demanding functions like management of attention)^9^. Although only a few studies have examined these three networks explicitly in GAD and with heterogenous results^10–12^, the respective brain regions associated with these networks have been investigated both during task-experiments and during the resting state (for reviews see^5,6^).

The most common technique for evaluating neural communication at the human network level is functional connectivity (FC) analysis, which involves assessing temporally dependent co-activation of anatomically separated brain regions^4^. Extant studies on FC in GAD have suggested abnormal relationships between specific brain regions, including the ventromedial prefrontal cortex (vmPFC)^13^, the insular cortex^6,14^, the amygdala^5,6,13,15,16^, and the dorsolateral prefrontal cortex (dlPFC)^13^. Additionally, analyses of functional magnetic resonance imaging (fMRI) data suggest that GAD is characterized by abnormal local responses in the dorsal anterior cingulate cortex (dACC)^17^ and the dorsomedial prefrontal cortex (dmPFC)^18^ in task-based experiments and by altered FC of the posterior cingulate cortex (PCC)^12^ and the temporal pole (TP)^16^ at rest. The aforementioned brain regions have been associated with numerous mental processes relevant to the psychopathology of GAD (described in Supplement), and many of these regions are key components of the DM, SN, and CEN.

To date, most studies on resting state FC in GAD have selectively interrogated relationships between subsets of brain regions, often relying purely on the null-hypothesis significance testing (NHST) framework^12–17^. While this form of frequentist inference requires several assumptions, one of them is particularly challenging in the context of neuroimaging: the conventional mass-univariate analysis unrealistically assumes uniform distribution across spatial units (i.e., voxels, regions). As effects across the brain tend to approximately follow a normal distribution, the conventional approach suffers from issues such as information loss, overfitting, and artificial dichotomization^19^. Further, parameter estimation in NHST stabilizes over large sample sizes^20^, but these samples are not readily obtainable in clinical populations. Even though more robust, non-parametric methods (e.g., threshold-free cluster enhancement^21^) have been developed within the frequentist framework, inherent limitations of NHST suggest the need for additional, if not alternative, ways of looking at the data.

Bayesian inference is an approach able to assess evidence in the data both for and against the experimental hypotheses, by allowing the researcher to assess for evidence of invariances as well as differences in a variable of interest^22^. In addition, instead of treating each spatial unit as an isolated entity, as in the conventional mass-univariate NHST analysis, Bayesian multilevel modeling integrates all spatial units into one holistic framework in which all the information is shared and leveraged through partial pooling^19^. The recent implementation of the multilevel Bayesian modeling matrix-based analysis program (MBA) in AFNI^23^ is one such example, which enables researchers to infer the probability of a research hypothesis, given the data, while overcoming the issue of multiplicity^24^.

In this preregistered study, we applied a Bayesian statistical framework to the analysis of resting state FC in GAD, with the addition of a frequentist analysis for a conventional comparison. We assessed the FC of brain regions previously implicated during task-experiments and during the resting state in GAD (vmPFC, dmPFC, dlPFC, dACC, insula, amygdala, PCC, TP) with respect to a focused set of hypotheses regarding potential group differences relative to healthy comparisons (HC) (Table 1). In addition to testing hypotheses stemming from the prior frequentist literature on GAD, the application of multilevel Bayesian modeling enabled us to more effectively address certain issues associated with NHST such as the problem of multiplicity and to evaluate observed relationships for convergence (i.e., to functionally “dissect” the data) across analytic approaches.

**Table 1:** *A-priori* **hypotheses about differences in functional connectivity between pre-defined regions of interest in generalized anxiety disorder relative to healthy comparisons.**

Region A	Region B	Hypothesis on FC
Posterior cingulate cortex	Ventromedial prefrontal cortex	Decreased^12^
Posterior cingulate cortex	Dorsomedial prefrontal cortex	Decreased^12^
Anterior insula	Dorsal anterior cingulate cortex	Decreased^39^
Dorsolateral prefrontal cortex	Amygdala	Increased^13,40^
Anterior insula	Ventromedial prefrontal cortex	Decreased^29^
Posterior-mid insula	Ventromedial prefrontal cortex	Decreased^29^
Amygdala	Dorsal anterior cingulate cortex	Decreased^15,41^
Amygdala	Temporal pole	Increased^16^
Amygdala	Ventromedial prefrontal cortex	Increased^42,43^
Amygdala	Anterior insula	Increased^44,45^
Amygdala	Posterior/mid insula	Increased^44,45^

Bilateral regions of interest (ROIs) were defined according to the label groupings from the Brainnetome atlas^38^: Posterior cingulate cortex, ventromedial prefrontal cortex, dorsomedial prefrontal cortex, dorsal anterior cingulate cortex, anterior insula (encompassing the agranular insula in entirety), posterior-mid insula (encompassing the granular and dysgranular insula in entirety), amygdala, dorsolateral prefrontal cortex, temporal pole. The corresponding IDs from the Brainnetome atlas defining each ROI are listed in the Supplement. References to previous literature our hypotheses were derived from are denoted in superscript numbers.

## Methods

The study hypotheses and data analysis plan were registered on the Open Science Framework before any of the study data was accessed or processed and all study data and analysis scripts are available online^25^.

### Participants

The study sample consisted of 58 participants (n_GAD_ = 29, n_HC_ = 29) matched on measured BMI and self-reported age. Three participants were excluded from further analysis due to excessive motion or signal outliers during their resting scan (see “Preprocessing” section), resulting in a final analysis sample of 27 GAD and 28 HC participants (Fig. S1). The diagnosis of GAD was verified by an experienced clinician administering the MINI neuropsychiatric interview^26^ according to the DSM-5 criteria^27^ of excessive anxiety occurring more days than not for at least 6 months, difficulty controlling the worry, consequent impairment in important areas of functioning not attributable to substance effects or other medical conditions, and three of the six key symptoms: restlessness, being easily fatigued, difficulty concentrating, irritability, muscle tension, or sleep disturbance. Additional GAD inclusion criteria were a currently elevated level of anxiety as evidenced by a GAD-7 questionnaire score greater than 10 out of 21 or an Overall Anxiety Severity and Impairment Scale (OASIS)^28^ score greater than 7 out of 20. Selected psychotropic agents (e.g., serotonergic/noradrenergic) were allowed so long as they were stably medicated (no changes within four weeks). We report data that was collected as part of a larger fMRI study that included an interoceptive perturbation task (isoproterenol infusion) performed after collection of the resting data presented here^29^. Since the larger study focuses on psychiatric disorders that predominantly occur in females (e.g., GAD, anorexia nervosa), the sample base for this investigation was also female-only. Further details on the aforementioned study can be found on the ClinicalTrials.gov registration (NCT02615119). All participants were administered the Patient Health Questionnaire depression module (PHQ-9)^30^, the GAD-7 questionnaire^31^, the OASIS^28^, the State-Trait Anxiety Inventory (STAI)^32^, and the Anxiety Sensitivity Index (ASI)^33^. HCs were required to be without any history of psychiatric illness per the MINI interview. The HC group was individually matched to the GAD group so that they would not differ significantly on body mass index (BMI) and age due to the known influence of the former on head motion^34^ and the latter on FC^35^. Further details on inclusion criteria and selection of participants (including CONSORT diagram) are listed in the Supplement.

The study was approved by the Western institutional review board and was conducted at the Laureate Institute for Brain Research. All participants provided written informed consent and received financial compensation for their study involvement.

### Image acquisition

Magnetic resonance images were obtained using two identical full-body 3.0 Tesla MR750 MRI scanners (GE Healthcare, Milwaukee, WI), equipped with an 8-channel head array coil (GE Healthcare, Milwaukee, WI). First, a T1-weighted image was acquired as an anatomical reference, followed by an 8-min resting-state scan using a single-shot gradient-recalled echo-planar imaging (EPI) sequence (see Supplementary Methods for details). Prior to the resting-state scan, participants were instructed to remain as still as possible, to keep their eyes open and fixated on a cross presented at the center of the screen, and to “clear your mind and do not think about anything in particular”.

### Data processing

#### Preprocessing

Preprocessing of fMRI data was conducted using AFNI 20.0.19 (RRID:SCR_005927)^23^ and Freesurfer 6.0.0 (RRID:SCR_001847)^36^. T1-weighted images were skull stripped and nonlinearly warped to Montreal Neurological Institute (MNI) 152 atlas space. Physiological noise effects (i.e., due to cardiac pulsatility and respiration) were regressed out using the RETROICOR method^37^ implemented in AFNI. Volumes censored due to too much motion or being signal outliers were interpolated using the previous and subsequent volume. Participants displaying excessive motion or signal outliers during their resting scan (i.e., > 30% volumes being censored because of motion or signal outliers) were excluded. See Supplement for further data preprocessing details.

#### Region of interest definition and data extraction

Based on a careful review of the fMRI literature on GAD we assessed FC between a total of nine regions of interest (ROIs). We then formulated a total of 11 *a-priori* hypotheses about FC between the nine pre-defined ROIs for examination (Table 1). To extract the data for each ROI, a mask was created by collapsing over the relevant labels of the Brainnetome atlas^38^, which provides a probabilistic cytoarchitectonic parcellation of the human brain. The average timeseries for each ROI was then extracted for each participant.

#### Statistical analyses

Using the timeseries of the nine ROIs we constructed a 9 × 9 correlation matrix for each participant. The relationship between ROIs was assessed using Pearson’s correlation. The resulting sampling distribution of Pearson’s *r* was normalized using the *Fisher r-to-z transformation* and the obtained z-scores were used in all further analyses.

### Bayesian modeling

A Bayesian multilevel model (BML)^24^ was applied to the data using the MBA program in AFNI, estimating the posterior probability of the effect being greater than 0 (*P*_+_). The BML was also used to explore all other possible region pairs that we did not hypothesize *a-priori* to be aberrant in GAD, and therefore left out of the FC analysis. The BML overcomes limitations of NHST in this context by (a) incorporating the interrelationships between region pairs into one model through partial pooling, (b) addressing the issue of multiplicity in the conventional NHST framework, (c) providing direct evidence for or against the effect of a region pair instead of assuming the null-hypothesis^46^, and (d) estimating the contribution of each individual brain region in the network relative to all other regions as a measurement of “relative importance”. Additionally, the BML inherently supports full result reporting and treats statistical evidence as a continuum instead of arbitrarily dichotomizing results as “significant” or “not significant”. While acknowledging that measures of uncertainty (e.g., confidence intervals) and effect sizes can also be derived in the NHST framework, results obtained with frequentist inference are often primarily evaluated by their *p*-value^47^. For a formal explanation of how the BML is used to estimate a posterior probability distribution please refer to Chen et al.^24^.

### Mass univariate analysis

Welch’s independent samples *t*-tests were used to test the null hypothesis that there was no difference in FC-scores between the two groups. To prevent the inflation of Type I errors (i.e., the problem of multiplicity) the results were Bonferroni corrected. The conservative Bonferroni correction was chosen over other frequent multiplicity correction methods in the field (i.e., family-wise error rate or false discovery rate) to improve the validity of the results and to facilitate a meaningful comparison with the BML approach. To decrease the likelihood of committing Type II errors, only the region pairs hypothesized to be aberrant in GAD (Table 1) were tested and consequently the Bonferroni corrected *α*-value for each hypothesis was adjusted based on how often the data of a ROI was used in multiple comparisons (for further details on multiplicity correction see Supplement). Finally, exploratory relationships between FC and symptom scores were examined using Pearson’s correlation, Bonferroni corrected for multiple comparisons, and corresponding Bayes Factors (BF) were calculated using the “BayesFactor” package in R.

### Ethical standards

The authors assert that all procedures contributing to this work comply with the ethical standards of the relevant national and institutional committees on human experimentation and with the Helsinki Declaration of 1975, as revised in 2008.

## Results

### Sample characteristics

Demographic and clinical information of the female study sample are summarized in Table 2. The participants did not differ significantly on age, BMI, or average head motion (see Supplement). As expected, the GAD group exhibited higher psychopathology scores on the PHQ-9, OASIS, GAD-7, STAI, and ASI questionnaires (Table 2).

**Table 2:** **Demographic and clinical characteristics of study sample.**

	GAD *n* = *27 females*	HC *n* = *28 females*	*∆M*	*95% CI*	*df*	*t*	*p*
Age (years)	26.2 ± 6.5	24.2 ± 5.1	− 1.99	[− 5.12, 1.18]	49.23	− 1.26	0.214
BMI (kg/m^2^)	25.9 ± 4.7	24 ± 3.2	− 1.86	[− 4.04, 0.32]	45.5	− 1.72	0.092
PHQ-9	11.5 ± 5	0.7 ± 1.1	− 10.8	[− 12.82, − 8.78]	28.37	− 10.96	< 0.001
OASIS	11 ± 2.2	1.1 ± 1.6	− 9.82	[− 10.87, − 8.78]	46.97	− 18.9	< 0.001
GAD-7	13.7 ± 3.4	1 ± 1.5	− 12.74	[− 14.19, − 11.29]	35.93	− 17.78	< 0.001
STAI-State	44.1 ± 9.3	24.7 ± 5.6	− 19.37	[− 23.59, − 15.15]	42.48	− 9.26	< 0.001
STAI-Trait	58 ± 6.7	28.2 ± 6.9	− 29.81	[− 33.51, − 26.12]	51.93	− 16.17	< 0.001
ASI-Total	28 ± 14.6	7.2 ± 4	− 20.71	[− 26.67, − 14.76]	29.74	− 7.1	< 0.001

All values presented as means ± standard deviation. Differences between group means (*∆M*) and corresponding 95% uncertainty intervals (CI) are reported. Welch’s independent samples *t*-tests were conducted to assess differences between the groups. Data for the STAI was missing from one HC participant. *GAD* generalized anxiety disorder, *HC* healthy comparison, *df* degrees of freedom, *BMI* body mass index, *PHQ-9* Patient Health Questionnaire-9, *OASIS* Overall Anxiety Severity and Impairment Scale, *GAD-7* 7-item generalized anxiety scale, *STAI* State-Trait Anxiety Inventory, *ASI* Anxiety Sensitivity Index.

### Resting-state fMRI analysis

#### Bayesian multilevel modeling

The results obtained from administering the BML^24^ to our data identified several region pairs with strong evidence for the group difference being greater (or less) than 0, most notably the vmPFC-PMI region pair (*P*_+_ = 0.98). The BML indicated strong evidence for a group difference for some of the region pairs that were also tested in the NHST model (i.e., dlPFC-amygdala, *P*_+_ = 0.96; vmPFC-AI, *P*_+_ = 0.95; PMI-amygdala, *P*_+_ = 0.95).

**Figure 1:**
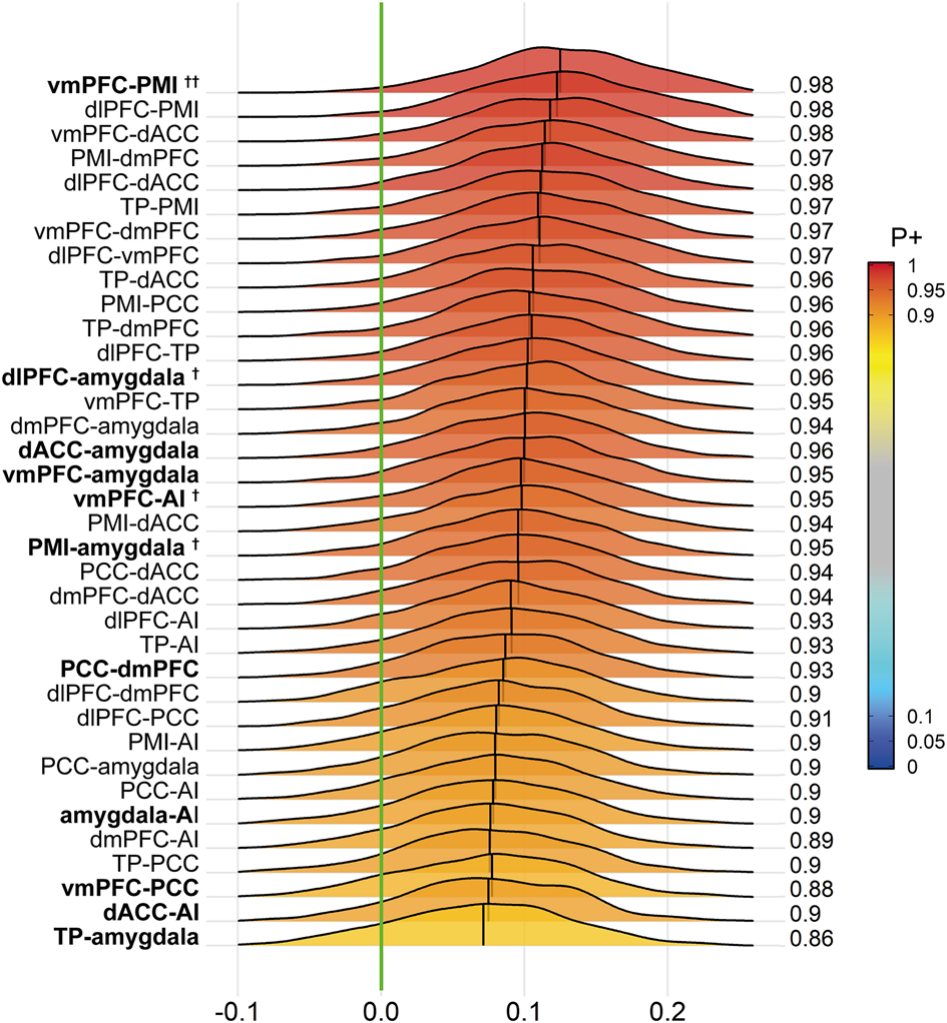
**Posterior density distributions of the difference in region-pair effect magnitudes between the two study groups as revealed by the Bayesian multilevel analysis.** The value at the right end of each curve indicates the posterior probability *P*_+_ for the group difference of the effect being greater than 0 (indicated by the vertical green line). The posterior probability *P*_+_ is additionally color-coded in the plane under each posterior density. The vertical black line in each distribution represents the mean effect difference between the two groups for each region pair. Bold font indicates region pairs included in the NHST analysis, with single daggers (^†^) indicating significance in the NHST analysis before, and two daggers (^††^) after, Bonferroni-correction for multiple comparisons. *vmPFC* ventromedial prefrontal cortex, *PMI* posterior-mid insula, *dlPFC* dorsolateral prefrontal cortex, *dACC* dorsal anterior cingulate cortex, *dmPFC* dorsomedial prefrontal cortex, *TP* temporal pole, *PCC* posterior cingulate cortex, *AI* anterior insula.

Interestingly, the BML analysis identified other region pairs to have reduced FC that were not hypothesized *a-priori* and were therefore not examined in the NHST analysis. These included the dlPFC-PMI, the vmPFC-dACC, the dmPFC-PMI, the dlPFC-dACC, the TP-PMI, and the vmPFC-dmPFC region pairs, all of which indicated high probabilities for a group difference. The complete results of the BML for all region pairs are illustrated in Fig. 1.

**Figure 2:**
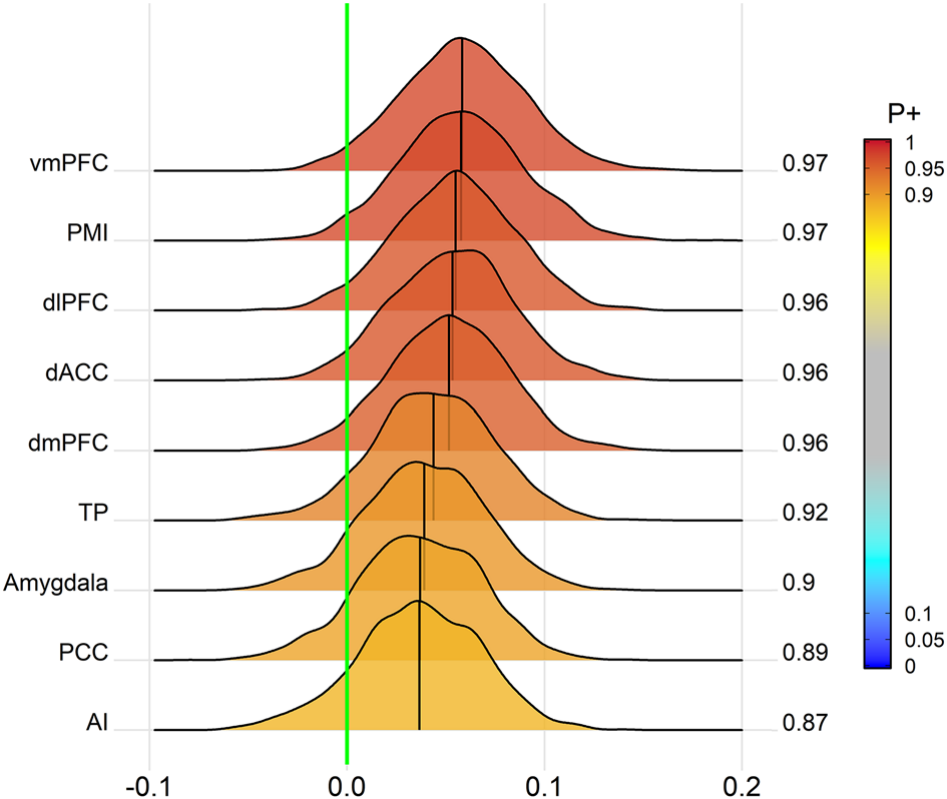
**Bayesian multilevel analysis reveals the vmPFC and PMI to have strong evidence for a difference in region effects between the study groups.** Posterior density distributions of the difference in region effects in the HC minus GAD contrast of the Bayesian multilevel model. The value at the right end of each curve indicates the posterior probability *P*_+_ for the group difference of the effect being greater than 0 (indicated by the vertical green line). The posterior probability *P*_+_ is additionally color-coded in the plane under each posterior density. The vertical black line in each distribution represents the mean difference in region effects (as Fishers’s *z*-score) between the two groups for each region in the model. *vmPFC* ventromedial prefrontal cortex, *PMI* posterior-mid insula, *dlPFC* dorsolateral prefrontal cortex, *dACC* dorsal anterior cingulate cortex, *dmPFC* dorsomedial prefrontal cortex, *TP* temporal pole, *PCC* posterior cingulate cortex, *AI* anterior insula.

The finding of the vmPFC-PMI region pair showing strong evidence for a group difference in the BML was reinforced by individual region effect estimates for the group comparisons: Both the vmPFC (*P*_+_ = 0.972) and the PMI (*P*_+_ = 0.972) showed the highest posterior probabilities for observing a region effect in the HC minus GAD contrast of all areas included in the BML. The complete list of region effects and their respective probabilities are shown in Fig. 2.

#### Mass-univariate analysis

**Figure 3:**
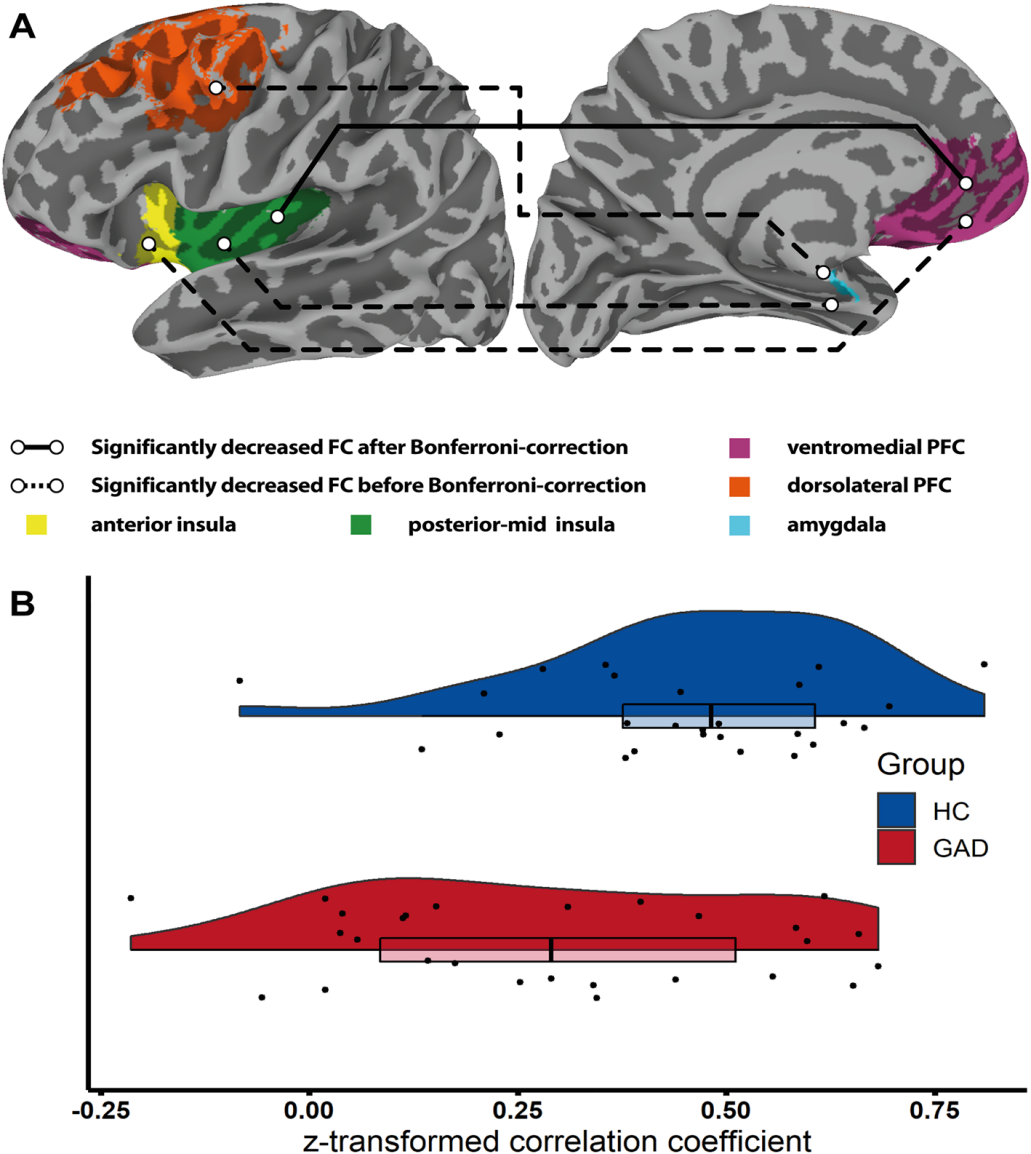
**Differences in resting-state FC between GAD and HC revealed by the frequentist analysis.** (**A**) The solid line indicates a significantly decreased FC between the PMI and vmPFC in GAD participants after multiple comparison correction using the Bonferroni method. Dotted lines indicate differences in FC between the PMI and amygdala, AI and vmPFC, and dlPFC and amygdala that did not remain statistically significant after Bonferroni correction. Each brain region, indicated by different colors, reflects the selected labels drawn from the Brainnetome atlas. (**B**) Raincloud plots of *Fisher r-to-z* transformed correlation coefficients between the PMI and the vmPFC BOLD-signal time series. *FC* functional connectivity, *GAD* generalized anxiety disorder, *HC* healthy comparison, *vmPFC* ventromedial prefrontal cortex, *PMI* posterior-mid insula, *AI* anterior insula, *dlPFC* dorsolateral prefrontal cortex.

Results from the conventional mass-univariate analysis revealed that participants in the GAD group had significantly lower FC compared to HCs between the vmPFC and PMI, the vmPFC and AI, the amygdala and PMI, and the amygdala and dlPFC region pairs. However, after Bonferroni-correction of all hypotheses tested, only the vmPFC and PMI result remained significant (Fig. 3). This finding is in line with the top result from the BML, that convergently identified the vmPFC-PMI region pair to have the highest probability for a group difference. Detailed results for all 11 hypotheses tested can be found in Table 3.

**Table 3:** **Results of the frequentist analysis of functional connectivity between selected region pairs.**

Region A	Region B	*M*_*GAD*_	*SE*_*GAD*_	*M*_*HC*_	*SE*_*HC*_	*∆M*	*95% CI*	*df*	*t*	*p*	*p*_*adj*_
PCC	vmPFC	0.81	0.04	0.86	0.03	0.05	[− 0.05, 0.15]	52.4	1.01	0.316	1
PCC	dmPFC	0.43	0.05	0.49	0.05	0.06	[− 0.07, 0.18]	53	0.85	0.399	0.797
AI	dACC	0.69	0.03	0.71	0.05	0.03	[− 0.08, 0.14]	44.3	0.48	0.636	1
dlPFC	Amygdala	0.20	0.04	0.30	0.03	0.11	[0.01, 0.2]	51.5	2.16	0.036^†^	0.215
AI	vmPFC	0.41	0.04	0.52	0.03	0.11	[0.01, 0.21]	52.5	2.18	0.034^†^	0.136
PMI	vmPFC	0.29	0.05	0.47	0.04	0.18	[0.06, 0.3]	48.9	2.94	0.005^††^	0.02^††^
Amygdala	dACC	0.18	0.03	0.26	0.04	0.07	[− 0.02, 0.17]	50.4	1.53	0.132	0.791
Amygdala	TP	0.45	0.03	0.52	0.03	0.07	[− 0.01, 0.16]	52.9	1.69	0.097	0.584
Amygdala	vmPFC	0.33	0.04	0.43	0.03	0.1	[0, 0.2]	52.5	1.99	0.052	0.314
Amygdala	AI	0.25	0.04	0.32	0.03	0.07	[− 0.02, 0.16]	46.5	1.58	0.12	0.719
Amygdala	PMI	0.33	0.03	0.43	0.03	0.1	[0.01, 0.19]	49.6	2.26	0.029^†^	0.171

Region pairs with significant differences in FC between the groups before multiplicity correction are indicated by a single dagger (^†^); the region pair with a significant difference in FC after multiplicity correction is indicated by double daggers (^††^). *M* mean, *SE* standard error of the mean, *∆M* differences between group means, *CI* confidence interval (uncertainty interval), *df* degrees of freedom, *PCC* posterior cingulate cortex*, vmPFC* ventromedial prefrontal cortex, *dmPFC* dorsomedial prefrontal cortex, *AI* anterior insula*, dACC* dorsal anterior cingulate cortex, *dlPFC* dorsolateral prefrontal cortex, *PMI* posterior-mid insula, *TP* temporal pole.

### Exploring the relationship between vmPFC-PMI connectivity and symptom scores

To investigate whether the reduced FC between the vmPFC and PMI, observed in both frequentist and Bayesian analyses, was related to greater psychopathology, we calculated Pearson’s *r* between vmPFC-PMI z-scores and clinical scores assessed by the following validated questionnaires: The GAD-7, the PHQ-9, the ASI, the STAI, and the OASIS. The correlation between vmPFC-PMI z-score and the ASI Total score was statistically significant only in the GAD group and before multiplicity correction but the Bayes Factor indicates that the presence of correlation between the vmPFC-PMI z-score and the ASI Total score was 3 times more likely than the absence of correlation (*r* = − 0.42, 95% CI [− 0.69, − 0.05], *p* = 0.029, *p*_*adj*_ = 0.175, BF = 3.03, Fig. S2). All other correlations with clinical scores were statistically non-significant even before multiplicity correction (Table 4) and their Bayes Factors indicated the absence of correlation.

**Table 4:** **Pearson’s correlation between vmPFC-PMI functional connectivity and clinical variables.**

	GAD	HC
vmPFC-PMI z-score—ASI total	*r* = − 0.42 95% CI [− 0.69, − 0.05] *p* = 0.029* *p*_*adj*_ = 0.175 *BF* = 3.03	*r* = 0.182 95% CI [− 0.21, 0.52] *p* = 0.353 *p*_*adj*_ = 1 *BF* = 0.59
vmPFC-PMI z-score—GAD-7	*r* = − 0.033 95% CI [− 0.41, 0.35] *p* = 0.872 *p*_*adj*_ = 1 *BF* = 0.42	*r* = 0.06 95% CI [− 0.32, 0.42] *p* = 0.762 *p*_*adj*_ = 1 *BF* = 0.43
vmPFC-PMI z-score—PHQ-9	*r* = − 0.075 95% CI [− 0.44, 0.31] *p* = 0.709 *p*_*adj*_ = 1 *BF* = 0.44	*r* = − 0.279 95% CI [− 0.59, 0.11] *p* = 0.15 *p*_*adj*_ = 0.902 *BF* = 0.98
vmPFC-PMI z-score—STAI state	*r* = − 0.061 95% CI [− 0.43, 0.33] *p* = 0.763 *p*_*adj*_ = 1 *BF* = 0.44	*r* = − 0.013 95% CI [− 0.39, 0.37] *p* = 0.949 *p*_*adj*_ = 1 *BF* = 0.42
vmPFC-PMI z-score—STAI trait	*r* = 0.05 95% CI [− 0.37, 0.42] *p* = 0.803 *p*_*adj*_ = 1 *BF* = 0.43	*r* = 0.205 95% CI [− 0.19, 0.54] *p* = 0.306 *p*_*adj*_ = 1 *BF* = 0.65
vmPFC-PMI z-score—OASIS	*r* = − 0.174 95% CI [− 0.52, 0.22] *p* = 0.385 *p*_*adj*_ = 1 *BF* = 0.57	*r* = − 0.125 95% CI [− 0.48, 0.26] *p* = 0.526 *p*_*adj*_ = 1 *BF* = 0.49

Pearson correlation coefficients are presented as test statistics along with their corresponding *p*-values (raw and adjusted for multiple comparisons using the Bonferroni method), 95% uncertainty intervals (CI), and Bayes Factors. *HC* healthy comparison participants*, GAD* participants with generalized anxiety disorder, *vmPFC* ventromedial prefrontal cortex, *PMI* posterior-mid insula, *BF* Bayes Factor, *GAD-7* 7-item generalized anxiety scale, *PHQ-9* Patient Health Questionnaire-9, *ASI* Anxiety Sensitivity Index, *STAI* State-Trait Anxiety Inventory*, OASIS* Overall Anxiety Severity and Impairment Scale. *p < 0.05.

## Discussion

In this preregistered study, we examined resting state FC in females with GAD relative to matched HCs to test a set of *a-priori* hypotheses using dual statistical frameworks: Bayesian multilevel modeling and NHST. Converging results from both analyses confirmed diminished FC between the PMI and the vmPFC in the GAD group compared to HCs. FC between these regions was associated with one clinically relevant trait measure, anxiety sensitivity, in the GAD group.

Examining the data using Bayesian multilevel modeling overcomes common problems with NHST^47^ (see Supplementary Discussion) while providing evidence for abnormal FC among region pairs that were not hypothesized *a-priori* and were therefore not tested in the NHST analysis. This included evidence of decreased FC among several regions including the dlPFC-PMI, the vmPFC-dACC, the dmPFC-PMI, the dlPFC-dACC, the TP-PMI, and the vmPFC-dmPFC. Thus, the application of both statistical frameworks and approaching neural connectivity data in GAD from two different statistical viewpoints yielded confirmatory results (most notably vmPFC-PMI) and provided indications of other relationships worth examining further.

Both the insula and the vmPFC are brain regions relevant to cognitive and emotional processing and have consequently been examined in previous investigations of the pathophysiology of anxiety disorders. Numerous studies have implicated the vmPFC in decision making^48^, generation and regulation of emotion^49^, and fear conditioning^50^. In GAD, the vmPFC has been previously associated with greater fear generalization^39^, which fits the clinical picture of excessive worry in individuals with the disorder^1^. Moreover, abnormal vmPFC functioning has most often been implicated in anxiety disorders in regards to its proposed role of inhibiting amygdala output^51^ (but see a different perspective by Myers-Schulz and Koenigs^52^). This seems reasonable considering the widely accepted view of the amygdala as a central hub for fear processing^53^. However, several lines of evidence show the need to distinguish between exteroceptive fear processing, which is most prominently mediated through the amygdala, and interoceptive fear processing, which is most prominently mediated through the insular cortex. For example, studies of individuals with bilateral amygdala lesions have shown a remarkable absence of anxiety or panic in response to exteroceptive fear stimuli (e.g., visual stimuli like snakes, spiders, or film clips)^54^ but experienced fear and panic evoked by interoceptive stimuli (e.g., carbon dioxide inhalation causing dyspnea or β-adrenergic agonist infusion causing palpitations)^55^.

Interoception is a construct tightly linked to the insular cortex (among other regions including the medial prefrontal cortex and amygdala)^56^ and encompasses the processing of internal body signals by the nervous system^57^. Models of interoceptive processing have suggested a posterior-to-anterior integration of interoceptive signaling within the human insula^58,59^ that is supported by its pattern of cytoarchitectonic organization with an agranular rostral and dysgranular/granular mid and posterior divisions across humans and primates^60,61^. Studies examining the functional organization of the insula implicate the AI in task maintenance^62^, attention control^63^, emotion^64^, and predictive processing^59^, which is in line with increased insula activity during emotional processing tasks in anxiety-prone individuals^65^. Recent results from our group collected in the same sample show blunted vmPFC activity during an interoceptive perturbation task (pharmacologic infusions of a fast-acting peripheral adrenaline analog resulting in cardiorespiratory modulation^29^), a method that has been reliably shown to activate the insula^66^.

The results from this current study suggest the implication of the vmPFC and insula as networked brain regions in the pathophysiology of GAD. More precisely, reduced vmPFC-PMI FC could support the idea that individuals with GAD may have difficulty exercising top-down regulation of emotion due to aberrant processing of bottom-up signals flowing through an interoceptive hub: the insula. This hypothesis is backed by our observations of vmPFC and PMI differences between HC and GAD, which were confirmed by both statistical approaches. While reduced vmPFCI-PMI FC at rest could partly be explained by increased sensitivity of the insula to interoceptive events in the GAD group, it seems plausible that impaired prefrontal regulation of negatively valenced interoceptive states plays a stronger role in this connection based on the observation of vmPFC hypoactivation during the aforementioned cardiorespiratory perturbation task in the same sample^29^. We also found that FC between the vmPFC and PMI was negatively associated with anxiety sensitivity, which is broadly defined as the fear of experiencing anxiety-related sensations especially those arising from within the body (e.g., heart palpitations or dyspnea)^67^. In a clinical context, this could mean that the smaller the correlated activity between the vmPFC and the insula at rest, the more likely patients are to experience internal body states as anxiety provoking. However, this interpretation is preliminary and other clinical scores were not correlated with vmPFC-PMI FC, suggesting that this relation might be specific to the anxiety sensitivity construct. Also, this result was statistically significant only before Bonferroni correction and while the Bayes factor indicated that a relationship between vmPFC-insula FC and anxiety sensitivity is likely, our dual statistical approach did not converge on this result. In conclusion, this finding provides some initial evidence of functional association between abnormal neural activity in the vmPFC and PMI and a transdiagnostic trait underlying the initiation and maintenance of pathological anxiety^68^.

Other results from the frequentist analysis indicated abnormal FC of the amygdala in GAD. Though contrary to our hypothesis, we observed decreased rather than increased FC between the amygdala and the PMI. The direction of this finding also contrasts with previous reports of an amygdala-insula resting state network in both anxious adults^69^ and adolescents^15^, but on the other hand aligns with other previous findings of reduced amygdala-insula FC^13^. Additionally, FC between the amygdala and the dlPFC was decreased, not increased, in our GAD sample. This finding was against our hypothesis that was based on previous literature^13^. Decreased FC between the amygdala and the dlPFC, which is a central node in the CEN, could be argued to reflect a dysfunctional management of attention (a key function of the CEN^9^) towards threat-related stimuli, which is a key clinical feature of GAD^70^. However, the overly general view of the amygdala as the central hub of fear processing is challenged by the absence of amygdala involvement in human fear extinction in a recent meta-analysis^71^, and heterogenous amygdala findings across reviews of neuroimaging literature in GAD^5,72^. While the results from our cross-sectional study might hint at the possibility that the role of the amygdala might not be as pivotal to the maintenance of GAD as expected, both amygdala-related findings (i.e., reduced FC for the PMI-amygdala and the dlPFC-amygdala in the GAD group) did not withstand correction for multiplicity and would therefore not be considered statistically significant using the NHST model framework. On the other hand, evaluation of the results from the BML indicated high probabilities for a group difference regarding those region pairs, raising the question whether overly rigorous multiplicity correction might have induced a type II error in our NHST-analysis of those brain regions. Viewing the data from a different, i.e., Bayesian, perspective thus strengthened the validity of our reduced amygdala FC findings, permitting us to discuss these results and consider their potential implications for GAD. Further insight into FC of the amygdala (and more generally, all of the selected ROIs) could be gained by employing seed-based whole-brain voxel wise FC analysis, a common approach to identify the networked connectivity of brain regions^73^. However, large datasets are required with this method to have sufficient statistical power and consequently, efforts have been made by the ENIGMA consortium to provide an analysis pipeline for employing seed-based FC analysis on pooled datasets from multicenter studies^74^ that can provide such large sample sizes.

Bayesian multilevel modeling further allowed us to investigate relationships in GAD that were not hypothesized *a-priori* with minimal risk of information loss. Our analysis identified high probabilities for decreased FC of the PMI with the dlPFC, the dmPFC, and the TP. Decreased functional coupling of the PMI and the dlPFC could be interpreted to reflect abnormal signaling of internal body systems to a key region for executive functions like working memory^75^ and attention^76^: aspects of cognition known to be impaired in anxiety^77,78^. The reduced PMI FC between both the dmPFC (a brain area known to be hyperactivated in GAD during emotional processing^17^ and at rest^79^), and the TP (an area implicated in social and emotional processing^80^), align well with a proposed model of the insula as an “integral hub” for detecting salient events, and for switching attention to these stimuli in preparation for regulatory (i.e., visceromotor) processing^81^. These additional findings suggest that the insula shows decreased functional coupling at rest with brain areas that have previously been found to show aberrant activity and/or connectivity in anxious individuals and whose functions are relevant to the clinical characteristics of GAD. However, this interpretation remains preliminary and requires causal examination in further experiments.

The Bayesian multilevel analysis also revealed diminished FC of the vmPFC-dmPFC region pair in GAD, two key components of the DMN^7^. This finding is consistent with previous reports of DMN alterations in GAD^12^, albeit decreased FC between the vmPFC and dmPFC has not been reported previously. These regions of the DMN are hypothesized to promote functions like processing of emotion and self-referential cognition^7^, which are impaired in GAD^82^. Lastly, the Bayesian analysis revealed reduced FC with the vmPFC and the dlPFC, which are key components, respectively, of the DMN and CEN networks^8^. Additionally, the GAD group exhibited decreased FC of both these regions with the dACC, a key node in the salience network and hypothesized to facilitate “switching” between the spontaneous cognition of the DMN^83^ and executive functioning of the CEN^8^. These results hint at the possibility that decreased FC between the vmPFC and dlPFC could be mediated by reduced functional coupling of these regions to the dACC.

## Limitations

Limitations of this study include a female-only sample with modest size (that is still above average compared to fMRI studies in recent years^84^), selected psychotropic medication allowance, and the methodological limitation that correlational analysis cannot determine the causality or directionality (i.e., responsible region) for impaired FC observed within region pairs (see Supplement for further discussion). The choice of brain regions we investigated was based on previous literature, but is not exhaustive. Other brain areas relevant to pathological mechanisms in GAD (e.g., thalamus^85^ or striatum^44,86^) should be investigated in future studies. As mentioned in the “Discussion”, testing FC differences between region pairs does not allow for network analysis as commonly employed in seed-based FC analysis across the whole brain. Our focus on females with GAD was based on the fact that females outnumber males with the disorder by a factor of two to one^87^, and that our sample was drawn from a larger study examining psychiatric disorders predominantly affecting females (e.g., anorexia nervosa and GAD). Future research is needed to establish whether our findings extend to males, i.e., whether sex differences in FC play a role in GAD. A recent mega-analysis found structural brain differences only in males with GAD but no general effect of GAD on brain structure^88^, indicating that a dynamic approach using functional MRI could provide better insight into the neurobiology of GAD.

## Conclusion

We leveraged the strengths of the Bayesian inference framework to convergently identify reduced FC between the vmPFC and the PMI in GAD and identified an association of this relationship with the anxiety sensitivity trait. Bayesian multilevel modeling allowed us to identify decreased FC between region pairs excluded by the frequentist analysis and other previously undescribed regions, emphasizing the utility of this method for probing the pathophysiological basis of psychiatric disorders. Future fMRI studies of resting state FC may benefit from a similar approach.

## Supplementary Information

Supplementary Information.

## Data Availability

All study data and scripts necessary to replicate the results of this study are available online on the Open Science Framework: https://osf.io/vf7s4/.
