# Supplementary material for: Reduced vmPFC-insula functional connectivity in generalized anxiety disorder: a Bayesian confirmation study
Source: Sci Rep. 2023 Jun 14;13:9626. doi: 10.1038/s41598-023-35939-2 (PMC10267153; doi:10.1038/s41598-023-35939-2)
Supplement: Supplementary file 1 — Supplementary Information. [file 41598_2023_35939_MOESM1_ESM.docx]

**SUPPLEMENT**

**Reduced vmPFC-insula functional connectivity in generalized anxiety disorder: a Bayesian confirmation study**

Jonas L. Steinhäuser^a,b^, cand. med.*, Adam R. Teed^a^, Ph.D., Obada Al-Zoubi^a,c^, Ph.D., René Hurlemann^d,e^, M.D., Ph.D., Gang Chen^f^, Ph.D., Sahib S. Khalsa^a,g^, M.D., Ph.D.*

^a^ Laureate Institute for Brain Research, Tulsa, Oklahoma, USA.

^b^ Division of Psychological and Social Medicine and Developmental Neurosciences, Faculty of Medicine, Technische Universität Dresden, Dresden, Germany.

^c^ Department of Electrical and Computer Engineering, University of Oklahoma, Tulsa, Oklahoma, United States

^d^ Department of Psychiatry, School of Medicine & Health Sciences, University of Oldenburg, Oldenburg, Germany

^e^ Research Center Neurosensory Science, University of Oldenburg, Oldenburg, Germany

^f^ Scientific and Statistical Computing Core, National Institute of Mental Health, Bethesda, Maryland, USA

^g^ Oxley College of Health Sciences, University of Tulsa, Tulsa, Oklahoma, United States

***Authors for correspondence:**Jonas Steinhäuser, E-mail: [Jonas.Steinhaeuser@tu-dresden.de](mailto:Jonas.Steinhaeuser@tu-dresden.de)
Sahib Khalsa, E-mail: [skhalsa@laureateinstitute.org](mailto:skhalsa@laureateinstitute.org)
Laureate Institute for Brain Research, 6655 South Yale Ave. Tulsa, OK 74136, USA

**Supplementary Introduction**

The brain regions hypothesized to exhibit abnormal functional connectivity (FC) in generalized anxiety disorder (GAD) are associated with a variety of mental processes including the regulation of emotion (e.g., ventromedial prefrontal cortex (vmPFC), amygdala)^1,2^, interoception (e.g., insula)^3,4^, attention (e.g., posterior cingulate cortex (PCC))^5^, executive functioning (e.g., dorsolateral prefrontal cortex (dlPFC))^6,7^, decision making (e.g., dorsal anterior cingulate cortex (dACC), vmPFC)^8,9^, working memory (e.g., dlPFC)^6,10^, processing of mental states (e.g., dorsomedial prefrontal cortex (dmPFC))^11^, and theory of mind (e.g., temporal pole (TP))^12^.

**Supplementary Methods**

Participants

Females with GAD and female HCs were recruited for this study from the Tulsa metropolitan area via advertisement in newspaper, radio, and social media outlets as well as via outpatient referral from the Laureate Psychiatric Clinic and Hospital. After completing the experiments, the final sample was created by matching participants on self-reported age and measured body mass index using the “MatchIt”^13^ library in R. The selection of participants is visualized in a CONSORT diagram (Figure S1) and further described in Teed et al. (2022)^14^. Pro re nata (PRN) medications were not a criterion for exclusion so long as participants were able to abstain from their use for at least two days prior to testing. Any history of a psychotic disorder or bipolar disorder led to exclusion from this study. Because of the cardiovascular implications of the pharmacological task employed as part of the larger study, participants previously diagnosed with cardiac or respiratory diseases were excluded, as well as those with comorbid panic disorder. Individuals with GAD are frequently comorbid with major depressive disorder (MDD) and other anxiety disorders^15,16^. Participants in the GAD group had the following psychiatric comorbidities: 11/27 MDD, 9/27 MDD and social anxiety disorder (SAD), 1/27 SAD, 1/27 MDD and post-traumatic stress disorder (PTSD), 1/27 MDD and alcohol use disorder (mild), 1/27 SAD and obsessive-compulsive disorder, 1/27 MDD, SAD, and agoraphobia, 1/27 MDD, SAD, and PTSD. Individuals in the GAD group reported taking the following psychoactive medication: 3/27 selective serotonin reuptake inhibitor (SSRI), 2/27 selective norepinephrine reuptake inhibitor (SNRI), 1/27 SSRI/SNRI, and 1/27 medicinal tetrahydrocannabinol.

MRI data acquisition

The T1-weighted image was acquired using a magnetization-prepared rapid gradient echo (MPRAGE) sequence with sensitivity encoding (SENSE)^17^ over the duration of 5 minutes and 40 seconds. The sequence-parameters for the T1-weighted image were: FOV = 240×192 mm, matrix = 256×256, 186 axial slices, slice thickness = 0.9 mm, 0.938×0.938×0.9 mm^3^ voxel volume, TR = 5 ms, TE = 2.012 ms, SENSE acceleration factor R = 2, flip angle = 8°, delay time = 1400 ms, inversion time = 725 ms, sampling bandwidth = 31.25 kHz. The parameters for the resting-state sequence (that also used SENSE) were: TR = 2000 ms, TE = 27 ms, R = 2, FA = 78°, FOV = 240 mm, 39 axial slices with 2.9 mm thickness with no gap, matrix = 96×96. The echo-planar imaging (EPI) images were reconstructed into a 128×128 matrix that produced 1.875×1.875×2.9 mm^3^ voxel volume. During the scan, respiration was recorded using a pneumatic belt placed around the torso. Heart rate was recorded using a photoplethysmograph with an infrared emitter placed under the pad of the participant’s finger.

**
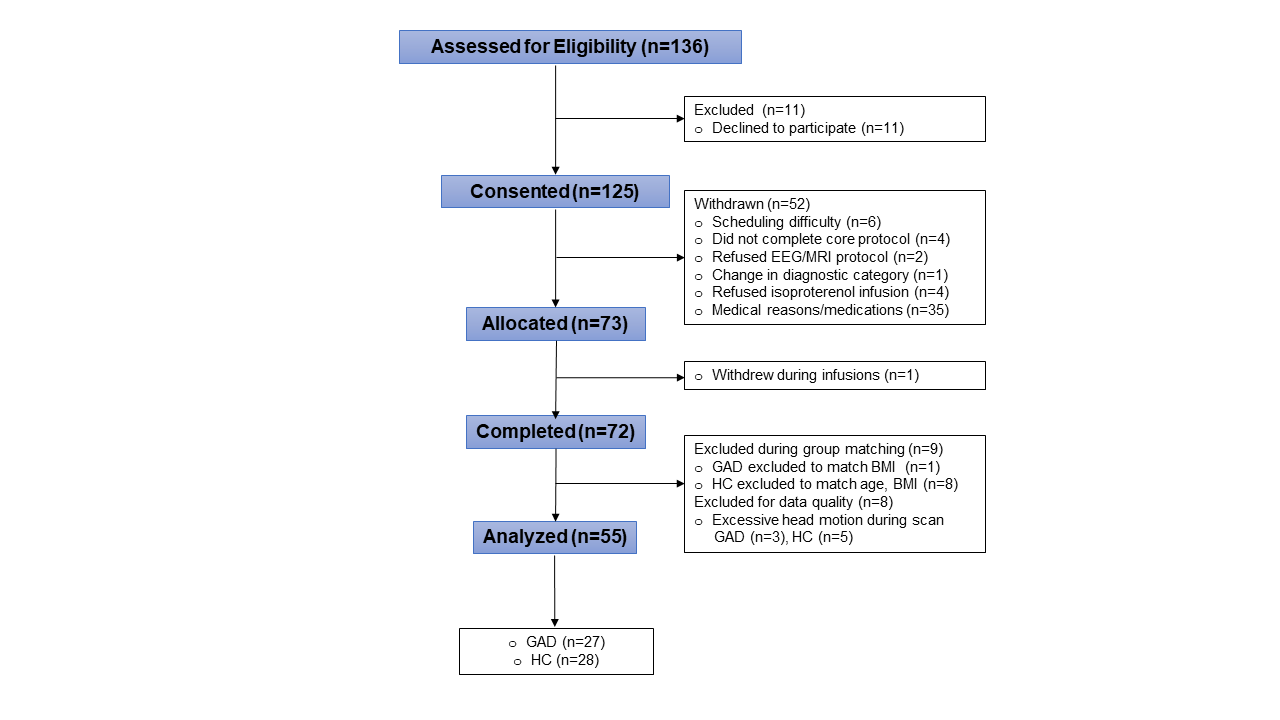
**

**Figure S1: CONSORT diagram of the isoproterenol (ISO) study and its resting state data analyzed for this investigation.** Adapted with permission from Teed et al. (2022)^14^. *HC* healthy comparison participants*, GAD* participants with generalized anxiety disorder, *ISO* isoproterenol, *rsfMRI* resting state functional magnetic resonance imaging

MRI data preprocessing

During preprocessing, white matter and ventricle masks were acquired to later regress out their signal from the data and the first three images of each participant’s timeseries were removed to ensure an equilibrium of fMRI signal. EPI volume signal was despiked using AFNI’s *3dDespike* program with default parameters. Slice timing correction was performed to account for interleaved slice acquisition. Anatomical images and EPI volumes for each participant were aligned to their EPI volume determined to have the minimum outliers according to a *Local Pearson Correlation Signed* cost function in AFNI. Datasets were blurred using a Gaussian kernel with full width half maximum of 4 mm. The time series of each voxel was scaled to a mean of 100, so that values could be interpreted as percentage change from the mean. Subsequently, voxel-values with a percentage increase of ≥ 100% were removed as outliers.

Region of interest definition

The Brainnetome^18^, a probabilistic atlas that allows for cytoarchitectonic parcellation of the brain, was used to define regions of interest (ROI) for our analysis of resting state data. The following IDs from the Brainnetome atlas were used to create a ROI analysis mask. PCC: *153, 154, 175, 176, 181, 182*; vmPFC: *41, 42, 45, 46, 47, 48, 49, 50, 187, 188*; dmPFC: *1, 2, 11, 12*; dACC: *179, 180, 183, 184*; Anterior insula (AI), encompassing the agranular insula in entirety: *165, 166, 167, 168*; Posterior/mid insula (PMI), encompassing the granular/dysgranular insula in entirety: *163, 164, 169, 170, 171, 172, 173, 174*; Amygdala: *211, 212, 213, 214*; dlPFC: *3, 4, 15, 16, 17, 18, 23, 24, 25, 26*; TP: *69, 70.*

Multiplicity correction in mass univariate analysis

Since some brain regions were included in more hypotheses than others, their data was used in multiple comparisons. The amygdala was included in six, the vmPFC in four, the AI in three, the PMI, PCC, and dACC in two, and the dmPFC, dlPFC, and TP in one comparison(s). Consequently, the significance level of the test results for each region pair were corrected based on how many comparisons the regions were included in.

**Supplementary Results**

Sample characteristics

The study groups did not differ with respect to average head motion during the resting state scan as indicated by the Wilcoxon rank sum test (*M_GAD_ =* 0.054, 95% CI [0.044, 0.065]; *M_HC_* = 0.057, 95% CI [0.046, 0.068]; *W* = 393, *p* = 0.809, ∆*M* = 0.003, 95% CI [-0.011, 0.017]).

**
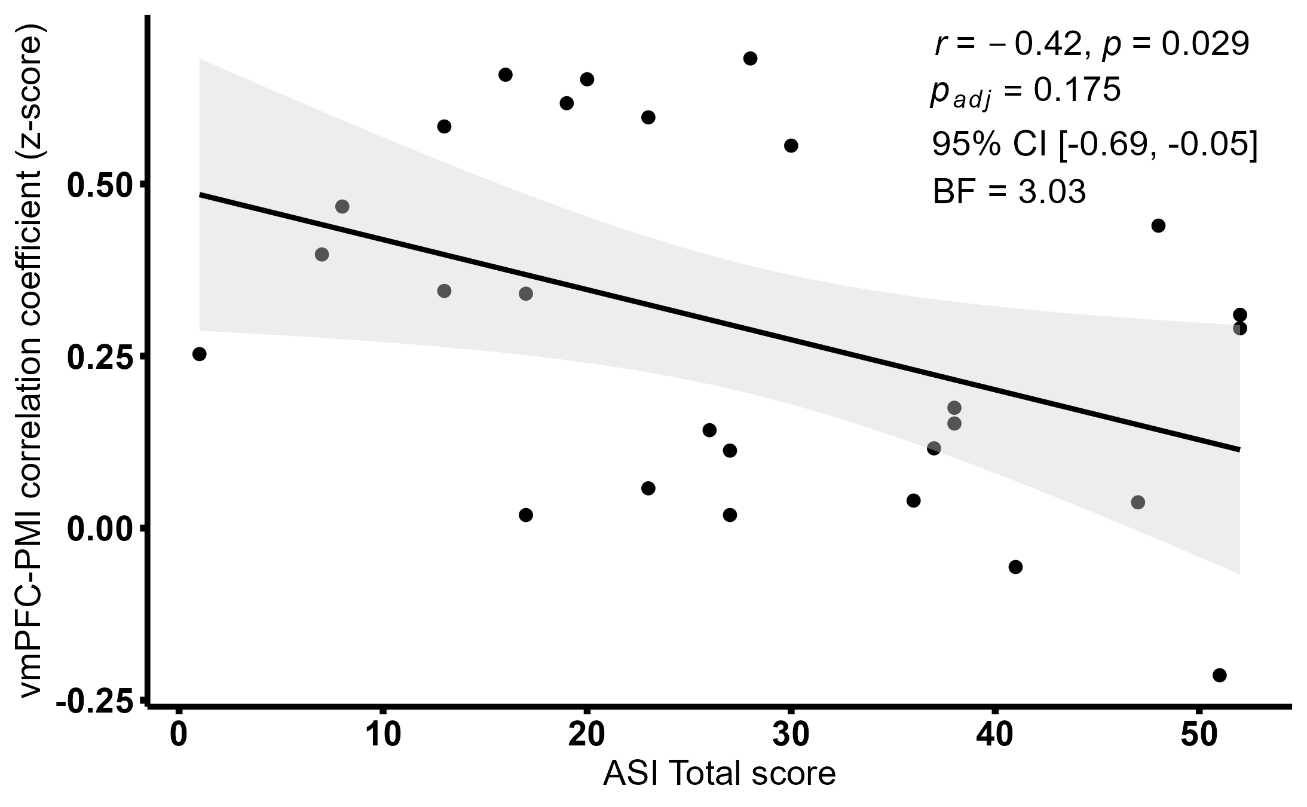
**

**Figure S2: Correlation between the z-transformed vmPFC-PMI correlation coefficient and the Anxiety Sensitivity Index (total) scores in the GAD group.** Scatterplot with fitted linear regression line for the vmPFC-PMI z-score and ASI-total scores for the GAD group only. Confidence interval for the regression line is displayed in light-gray. Pearson correlation coefficient, corresponding raw and Bonferroni corrected *p*-values and Bayes Factor are reported as test statistics. *GAD* generalized anxiety disorder, *vmPFC* ventromedial prefrontal cortex, *PMI* posterior-mid insula, *BF* Bayes Factor

**Supplementary Discussion**

Shortcomings of frequentist analysis

While the frequentist analysis in convergence with the BML identified the vmPFC and PMI region pair to exhibit decreased functional coupling in our analysis, the NHST framework as a whole has faced growing criticism^19–21^. Conceptually, NHST results are often misinterpreted (or misunderstood) given that they assess only whether the experimental result observed (e.g., that two groups differ on a variable of interest) is too unlikely to maintain an assumption that the null hypothesis is true^19,22^. This assumption precludes the ability to distinguish between results that provide evidence of the absence of an effect from null results yielded merely from a lack in statistical power. Additionally, using the same data to test multiple hypotheses in the NHST framework arbitrarily inflates the type I error^23^, resulting in a variety of methods to adjust for this problem of multiplicity^24,25^.

Limitations

Our approach is not able to determine whether or not altered FC between region pairs is mediated by a third brain region. Analysis of resting state fMRI data has received growing criticism regarding potential confounds including, most recently, the discovery of “resting state physiological networks” (i.e., physiologically driven FC resembling previously reported neural networks)^26^. We addressed this concern by recording and regressing out signals attributed to respiration or cardiac pulsatility^27^. A recent paper also identified substantial variability of fMRI results across many teams analyzing the same data set^28^, underlining the need for standardized fMRI analysis measures. To improve the reproducibility of our findings, we followed several of the recommended steps including 1) applying different statistical approaches to the data yielding largely converging results, 2) pre-registering our hypotheses and statistical approaches before analyzing any study data, 3) reporting results of all analyses conducted, even if they did not reach statistical significance, and 4) publicly sharing the code of our data processing pipeline and statistical analysis^29^.

**References**

1. Phelps, E. A. Emotion and Cognition: Insights from Studies of the Human Amygdala. *Annu. Rev. Psychol.* **57**, 27–53 (2006).

2. Hiser, J. & Koenigs, M. The Multifaceted Role of the Ventromedial Prefrontal Cortex in Emotion, Decision Making, Social Cognition, and Psychopathology. *Biol. Psychiatry* **83**, 638–647 (2018).

3. Craig, A. D. How do you feel? Interoception: the sense of the physiological condition of the body. *Nat. Rev. Neurosci.* **3**, 655–666 (2002).

4. Khalsa, S. S. *et al.* Interoception and Mental Health: A Roadmap. *Biol. Psychiatry Cogn. Neurosci. Neuroimaging* **3**, 501–513 (2018).

5. Leech, R. & Sharp, D. J. The role of the posterior cingulate cortex in cognition and disease. *Brain* **137**, 12–32 (2014).

6. Petrides, M. The role of the mid-dorsolateral prefrontal cortex in working memory. *Exp. Brain Res.* **133**, 44–54 (2000).

7. Mansouri, F. A., Tanaka, K. & Buckley, M. J. Conflict-induced behavioural adjustment: a clue to the executive functions of the prefrontal cortex. *Nat. Rev. Neurosci.* **10**, 141–152 (2009).

8. Bechara, A., Tranel, D. & Damasio, H. Characterization of the decision-making deficit of patients with ventromedial prefrontal cortex lesions. *Brain* **123**, 2189–2202 (2000).

9. Bush, G. *et al.* Dorsal anterior cingulate cortex: A role in reward-based decision making. *Proc. Natl. Acad. Sci.* **99**, 523–528 (2002).

10. Barbey, A. K., Koenigs, M. & Grafman, J. Dorsolateral prefrontal contributions to human working memory. *Cortex* **49**, 1195–1205 (2013).

11. Bzdok, D. *et al.* Segregation of the human medial prefrontal cortex in social cognition. *Front. Hum. Neurosci.* **7**, 232 (2013).

12. Gallagher, H. L. & Frith, C. D. Functional imaging of ‘theory of mind’. *Trends Cogn. Sci.* **7**, 77–83 (2003).

13. Ho, D., Imai, K., King, G. & Stuart, E. A. MatchIt: Nonparametric Preprocessing for Parametric Causal Inference. *J. Stat. Softw.* **42**, 1–28 (2011).

14. Teed, A. R. *et al.* Association of Generalized Anxiety Disorder With Autonomic Hypersensitivity and Blunted Ventromedial Prefrontal Cortex Activity During Peripheral Adrenergic Stimulation: A Randomized Clinical Trial. *JAMA Psychiatry* **79**, 323–332 (2022).

15. Nutt, D. J., Ballenger, J. C., Sheehan, D. & Wittchen, H.-U. Generalized anxiety disorder: comorbidity, comparative biology and treatment. *Int. J. Neuropsychopharmacol.* **5**, 315–325 (2002).

16. Noyes, R. Comorbidity in Generalized Anxiety Disorder. *Psychiatr. Clin. North Am.* **24**, 41–55 (2001).

17. Pruessmann, K. P., Weiger, M., Scheidegger, M. B. & Boesiger, P. SENSE: sensitivity encoding for fast MRI. *Magn. Reson. Med.* **42**, 952–962 (1999).

18. Fan, L. *et al.* The Human Brainnetome Atlas: A New Brain Atlas Based on Connectional Architecture. *Cereb. Cortex* **26**, 3508–3526 (2016).

19. Nickerson, R. S. Null hypothesis significance testing: A review of an old and continuing controversy. *Psychol. Methods* **5**, 241–301 (2000).

20. Greenland, S. *et al.* Statistical tests, P values, confidence intervals, and power: a guide to misinterpretations. *Eur. J. Epidemiol.* **31**, 337–350 (2016).

21. Wasserstein, R. L. & Lazar, N. A. The ASA Statement on *p* -Values: Context, Process, and Purpose. *Am. Stat.* **70**, 129–133 (2016).

22. Chen, G. *et al.* Handling Multiplicity in Neuroimaging Through Bayesian Lenses with Multilevel Modeling. *Neuroinformatics* **17**, 515–545 (2019).

23. Tukey, J. W. The Philosophy of Multiple Comparisons. *Stat. Sci.* **6**, 100–116 (1991).

24. Curran-Everett, D. Multiple comparisons: philosophies and illustrations. *Am. J. Physiol.-Regul. Integr. Comp. Physiol.* **279**, R1–R8 (2000).

25. Bender, R. & Lange, S. Adjusting for multiple testing--when and how? *J. Clin. Epidemiol.* **54**, 343–349 (2001).

26. Chen, J. E. *et al.* Resting-state “physiological networks”. *NeuroImage* **213**, 116707 (2020).

27. Glover, G. H., Li, T. Q. & Ress, D. Image-based method for retrospective correction of physiological motion effects in fMRI: RETROICOR. *Magn. Reson. Med.* **44**, 162–167 (2000).

28. Botvinik-Nezer, R. *et al.* Variability in the analysis of a single neuroimaging dataset by many teams. *Nature* **582**, 84–88 (2020).

29. Steinhäuser, J., Teed, A. & Khalsa, S. Correlated activity in generalized anxiety disorder - a resting-state fMRI approach. (2020) doi:10.17605/OSF.IO/J29QV.
